# Disorders of Consciousness: Painless or Painful Conditions?—Evidence from Neuroimaging Studies

**DOI:** 10.3390/brainsci6040047

**Published:** 2016-10-08

**Authors:** Francesca Pistoia, Simona Sacco, Janet Stewart, Marco Sarà, Antonio Carolei

**Affiliations:** 1 Neurological Institute, Department of Biotechnological and Applied Clinical Sciences, University of L’Aquila, L’Aquila 67100, Italy; simona.sacco@cc.univaq.it (S.S.); antonio.carolei@univaq.it (A.C.); 2 Psychology Division, School of Natural Sciences, University of Stirling, Scotland FK8, UK; janet@bollati.info; 3 Post-Coma Intensive Rehabilitation Care Unit, San Raffaele Hospital, Cassino 03043, Italy; marco.sara@sanraffaele.it

**Keywords:** consciousness, neuroimaging, pain, vegetative state, unresponsive wakefulness syndrome, minimally conscious state

## Abstract

The experience of pain in disorders of consciousness is still debated. Neuroimaging studies, using functional Magnetic Resonance Imaging (fMRI), Positron Emission Tomography (PET), multichannel electroencephalography (EEG) and laser-evoked potentials, suggest that the perception of pain increases with the level of consciousness. Brain activation in response to noxious stimuli has been observed in patients with unresponsive wakefulness syndrome (UWS), which is also referred to as a vegetative state (VS), as well as those in a minimally conscious state (MCS). However, all of these techniques suggest that pain-related brain activation patterns of patients in MCS more closely resemble those of healthy subjects. This is further supported by fMRI findings showing a much greater functional connectivity within the structures of the so-called pain matrix in MCS as compared to UWS/VS patients. Nonetheless, when interpreting the results, a distinction is necessary between autonomic responses to potentially harmful stimuli and conscious experience of the unpleasantness of pain. Even more so if we consider that the degree of residual functioning and cortical connectivity necessary for the somatosensory, affective and cognitive-evaluative components of pain processing are not yet clear. Although procedurally challenging, the particular value of the aforementioned techniques in the assessment of pain in disorders of consciousness has been clearly demonstrated. The study of pain-related brain activation and functioning can contribute to a better understanding of the networks underlying pain perception while addressing clinical and ethical questions concerning patient care. Further development of technology and methods should aim to increase the availability of neuroimaging, objective assessment of functional connectivity and analysis at the level of individual cases as well as group comparisons. This will enable neuroimaging to truly become a clinical tool to reliably investigate pain in severely brain-injured patients as well as an asset for research.

## 1. Disorders of Consciousness: An Overview

Disorders of consciousness (DOCs) is an umbrella term for coma, unresponsive wakefulness syndrome or vegetative state (UWS/VS) and minimally conscious state (MCS) [1]. In the case of coma, brainstem arousability is lost as a consequence of a lesion in the ascending reticular system or due to diffuse hemispheric damage. The patient in a condition of a coma is therefore neither awake nor aware [2]. In UWS/VS, brainstem arousability is preserved, but there is no sign of consciousness. The patient is considered to be awake but not aware [3]. Finally, patients in an MCS show fluctuating signs of consciousness: they are awake and also show awareness to a degree that can vary both between individuals and within the same individual over time [4]. Given the heterogeneity of their behavioural responses, this group is now further categorised as MCS+ and MCS− [5]. The former shows high-level behavioural responses such as command following, intelligible verbalisations or non-functional communication, while the latter shows only low-level behavioural responses such as visual pursuit, localisation in response to noxious stimulation or contingent behaviour such as appropriate smiling or crying to emotional stimuli [5]. The regaining of specific purposeful behaviours, such as the ability for functional object use and functional communication, denotes the emergence from the MCS towards a fully conscious state. A further subgroup of patients with DOCs is one composed of individuals that are completely unresponsive from a behavioural point of view but show covert consciousness when investigated by means of advanced neuroimaging or electrophysiological techniques [5,6]. There is evidence of wide dissociation of motor and cognitive function in these patients, but, at present, there is no agreed label to properly distinguish them as a category [7]. They were initially referred to as being in a *functional locked-in state* as they seem able to perform specific mental imagery tasks (such as imagining playing tennis or moving around their home) without the possibility of conveying mental activity through overt behaviours [5]. The term *cognitive motor dissociation* was later introduced to highlight the dissociation of measured bedside behaviour and laboratory investigations [7]. This condition, characterised by functional Magnetic Resonance Imaging (fMRI) or electrophysiological evidence of command-following despite the total absence of behavioural responsiveness, usually follows a widespread brain injury and sometimes represents a phase of recovery from UWS/VS [7,8,9]. Specific damage to motor thalamocortical fibres has recently been indicated as a biomarker of *cognitive motor dissociation*, as motor execution, unlike motor imagery, requires the proper working of an excitatory coupling between the thalamus and the primary motor cortex [10]. This state must be differentiated from *structural locked-in syndrome*, where a specific ventral brainstem lesion is responsible for a condition of motor entrapment due to the interruption of corticospinal and corticobulbar motor pathways [9,11]. By definition, these patients show preserved consciousness (after the acute phase of brain damage) and unaffected cognitive abilities, as the primary injury does not involve the supratentorial brain regions [9,11]. However, despite this traditional view, recent evidence shows that most patients with *structural locked-in syndrome* (LIS) may also experience cognitive dysfunctions including motor imagery defects and emotional disturbances, suggesting that these additional symptoms may contribute to the frequent confusion in diagnosis between patients with DOCs and *structural LIS* [12,13,14,15,16]. More recent findings also suggest that chronic patients with *structural LIS* react to the sudden interruption of motor pathways showing a later selective cortical loss [16]. Finally, further confusion may arise when patients with a structural LIS show additional remote brain lesions beyond the pontine one. This condition is more likely to occur after severe traumatic brain injuries causing scattered brain lesions both at a supratentorial and subtentorial level. In these cases, a neuropathological overlap between DOCs and LIS may lead to a variable combination of functional and structural motor entrapment [1].

### 1.1. Consciousness: Still a Hard Problem

Although consciousness is at the centre of human experience and enables our interaction with the world, it largely remains a mystery. The study of consciousness requires a recognition of its multi-level nature. The height of human consciousness involves an awareness of past, present and future, of abstract concepts and of matters which go well beyond the individual in the here and now. This level of functioning also involves processes such as reasoning, memory and language. On the other hand, the most basic level of consciousness, that which Damasio calls “core consciousness”, can be presumed to be a biologically simpler phenomenon, which is a prerequisite of higher levels of consciousness [17]. Whether it is the impairment of this core consciousness which is being referred to when we talk about UWS/VS is still questionable [17]. However, most of the recent studies investigating the presence of preserved islands of consciousness in brain-injured patients were exclusively aimed at revealing the presence of a covert higher-level reflective self-awareness through the use of fMRI associated with specific cognitive tasks [18,19,20,21]. For instance, during fMRI scanning, patients are asked to imagine playing tennis or moving around their home or to listen to factually correct vs. factually incorrect sentences, and their fMRI patterns are compared to those obtained from healthy subjects [18,19,20,21]. When the patterns of patients significantly resemble those of healthy subjects, the conclusion is that behaviourally unresponsive patients are, indeed, covertly conscious but unable to convey their preserved mental performances into perceived motor outputs, due to the aforementioned cognitive motor dissociation [7,18,19,20,21]. To date, these studies seem to be the most promising way to reach a better understanding of the pathophysiology of DOCs: however, one should remember that processing a stimulus does not necessarily imply being conscious of it. In fact, as suggested by Damasio, consciousness exists in the relationship between an organism and environmental stimuli in the feeling of feeling continuous experience, which underlies subjectivity [17,22]. All of these elements raise questions about what kind of consciousness should really be considered minimal [23]. In this respect, a recent comparison between unitary (all-or-none) and non-unitary (gradual or continuous) models of consciousness suggested that both kinds of models are compatible with the current definitions of UWS/VS and MCS. Evidence from neuroimaging studies supports the view of a non-unitary consciousness [23] and places patients with DOCs along a clinical continuum implying several gradients of consciousness. However, to date, this evidence does not enable definitive conclusions about the nature of consciousness and the mechanisms of its impairment.

In fact, the hardest problem we have to face when investigating consciousness and its disorders lies with our poor knowledge about its neural correlates. Over the past century, observations of patients with focal brain lesions allowed us to infer the anatomical organisation of the human brain and to identify the distinctive neural substrates of the main human abilities such as motor function, language, emotion, decision-making and attention. However, consciousness seems to escape this model as there is a great variability of brain lesions, which are associated with consciousness impairment and no linear relationship between the extent and localisation of brain damage and the ensuing disorder of consciousness. Consciousness can be regarded as an emergent phenomenon that is not the product of a single cortical area but the result of large-scale dynamics within widespread neural networks. Recent neuroimaging evidence has suggested that UWS/VS should in fact be considered a global disconnection syndrome in which higher order association cortices are functionally disconnected from primary cortical areas [24,25]. This is in line with functional imaging data showing that focal lesions, beyond interfering with the functioning of a single area, can also produce specific patterns of altered functional connectivity among distant regions of the cortex [26]. This impaired connectivity, associated with the functional isolation of parts of the brain, may be responsible for lack of recovery of consciousness in a subgroup of brain-injured patients [27,28,29]. Alternative results have been reported by other studies investigating the presence of specific patterns of brain damage in patients with DOCs [30,31]. One of these studies revealed the presence of extensive white matter lesions in patients with post-anoxic injury, with the largest lesions observed in the frontal and occipital lobes, demonstrating that white matter involvement, even in alternatives sites with respect to those traditionally considered, plays a crucial role in the impairment of consciousness following severe brain injuries [30]. This observation overturns the traditional view about the prevailing involvement of grey matter in post-anoxic damage, due to the higher energy demand of grey matter and its higher density of glutamate receptors, and suggests investigating both grey and white matter in future neuroimaging studies [30]. Other studies focused on the possible activation of high-level cortical areas in patients with DOCs in response to specific cognitive paradigms, thus stressing the role of a neuropathological overlap between grey and white matter lesions and their combined action in causing consciousness impairment [20,31].

All of these elements suggest that, although far from localising consciousness in the human brain, evidence from functional neuroimaging studies is certainly helping to disentangle the neural substrates of consciousness and to improve our ability to distinguish UWS/VS, MCS and functional locked-in state. Studies looking at responses to noxious stimuli are also playing an important role in this research as the degree of cortical engagement during noxious stimulation differs among patients with increasing levels of consciousness [32].

### 1.2. Emerging Neuroimaging Evidence about Covert Consciousness

Patients with UWS/VS or in an MCS have, by definition, suffered severe brain damage leading to a loss of consciousness and to concomitant neurological impairments. This means that each patient’s neurological picture tends to be complex and highly individual. Along with compromised consciousness, concomitant impairments may prevent patients from revealing their true level of consciousness or communicating pain. These include aphasia, low or fluctuating levels of arousal, severe spasticity or rigidity and a combination of other motor and cognitive symptoms as a result of multiple focal lesions or diffuse brain damage. We recently suggested that in some cases the behavioural unresponsiveness of patients is the consequence of the variable combination of sub-syndromes involving the pyramidal and extrapyramidal tracts, brainstem pathways and cortical areas [33]. Accurate diagnosis is notoriously challenging in patients with DOCs, with a reported misdiagnosis rate of around 40% [34]. The high occurrence of bedside underestimation of consciousness in these patients may be partly due to these concomitant impairments limiting the behavioural repertoire [33]. Indeed, in a small percentage of patients behaviourally diagnosed as having UWS/VS, neuroimaging has uncovered previously undetected functional communication, in the form of willful brain activity in response to verbal instructions [18,19,20,21,35]. More routine use of neuroimaging investigations may therefore help to bring to light cases of functional locked-in syndrome [9]. For these reasons, multimodal assessment is of particular relevance for this group of patients; a combination of bedside evaluation and functional neuroimaging techniques provides more insight into the patient’s condition than behavioural observation alone. The gold standard assessment of patients with DOCs should include careful and frequent clinical evaluation using the Coma Recovery Scale—Revised, which has good reliability for distinguishing between UWS/VS and MCS and for identifying emergence from MCS, along with neuroimaging and neurophysiological assessment [36,37].

### 1.3. Nociception and Pain

Central to the issue of pain in DOCs is the important distinction between nociception and pain. Nociception is a physiological term describing the neural mechanisms of encoding and processing an actually or potentially tissue-damaging event [38]. Pain, on the other hand, is “an unpleasant sensory and emotional experience associated with actual or potential tissue damage or described in terms of such damage” [38]. This distinction is important because, although often integrated, (nociceptive pain) each can occur without the other. A host of pain-related phenomena illustrate that pain is not necessarily proportionate to the degree of tissue damage taking place. For instance, pain can be perceived from amputated limbs or can occur without any evidence of physical damage, as is sometimes the case with fibromyalgia, low back pain or headaches. In cases of thalamic pain, no peripheral nociception is responsible for the suffering [39]. Conversely, there are circumstances in which pain seems to be absent or inhibited despite severe damage to tissues that are served by nociceptors. The subjective nature of pain has long been recognized, and there is now wide acceptance that it can be influenced by biological, cognitive, emotional, social and behavioural factors.

### 1.4. Behavioural Assessment of Pain and Successful Management

Responses to noxious somatosensory stimuli in individuals with DOCs are of clinical, scientific, therapeutic and ethical importance, first and foremost in guiding appropriate pain management and optimizing physical comfort [32]. Conscious, responsive subjects can usually provide subjective reports of their pain and lead decisions about its management. In the case of patients with DOCs this is not possible, while there are many potential sources of pain due to multiple injuries, medical complications or comorbidities [40,41]. In these conditions, pain management is very challenging. Patients have the right to adequate pain relief, but chronic over-medication may have toxic effects and over-sedation could mask signs of recovery of consciousness and compromise rehabilitation [42]. In this respect, the recent introduction of the Nociception Coma Scale—Revised has provided a useful tool to capture signs of pain during the behavioural assessment of patients with DOCs [43,44]. This tool is based on the observation of motor, verbal, and mimicry in response to painful stimulations. Its main limitation is that it is a behavioral motor-dependent scale with the ensuing consequence that subtle signs of pain in patients with extreme motor restrictions are at risk of being underestimated or misdiagnosed. Other behavioural scales have been widely used to assess pain in non-communicative patients such as the The Behavioral Pain Scale, the Critical Care Pain Observation Tool and the Scale of Behavior Indicators of Pain [45,46,47]. However, all of these tools show relevant limitations, which prevent us from drawing definitive conclusions when assessing nociception in this challenging group of patients. 

Recently, a European survey investigated beliefs concerning the experience of pain in patients with DOCs and found that 96% of the medical doctors and 97% of the paramedical professionals thought that patients in an MCS can feel pain [48]. When asked whether they thought that patients with UWS/VS could feel pain, 56% of the medical doctors and 68% of the paramedical professionals answered affirmatively [48]. This particular survey suggested effects of profession, sex, age and religion on such beliefs, highlighting the need for evidence-based medical guidelines for pain recognition and management. Neuroimaging techniques certainly provide a promising tool to improve both diagnosis and prognosis for patients with impaired consciousness and to contribute to more reliable identification and management of pain.

## 2. Emerging Neuroimaging Evidence about the Experience of Pain in Behaviourally Unresponsive Patients

The central nociceptive system in healthy subjects, known as the pain matrix, includes the primary (S1) and secondary (S2) somatosensory cortices, the lateral thalamus, the insular cortex, the anterior cingulate cortex (ACC), the dorsolateral prefrontal cortex (DLPFC), and the parietal cortex. Specifically, S2 and S2 cortices, the lateral thalamus, and the posterior insula belong to the lateral neuronal network that encodes sensory-discriminative information while the anterior insula, the ACC, and the prefrontal cortex belong to a medial network that encodes affective-cognitive information [49]. Several neuroimaging studies have investigated the processing of noxious somatosensory stimuli in patients with DOCs and all concluded that these areas are not jointly recruited during painful stimulations (Table 1). Laureys and colleagues performed positron emission tomography (PET) scanning on 15 patients with UWS/VS while they underwent high-intensity electrical stimulation of the median nerve. Changes in regional Cerebral Blood Flow (rCBF) were detected in the midbrain, contralateral thalamus and primary somatosensory cortex in all the patients, indicating the selective involvement of these structures during the noxious stimulation [50]. In contrast to healthy controls, no stimulus-induced activation was observed in the secondary somatosensory cortex, bilateral insula, caudal and rostral anterior cingulate and bilateral posterior parietal cortices. Indeed, functional connectivity analysis showed that the primary somatosensory cortex was functionally disconnected from the higher order associative areas which are thought to be necessary for conscious awareness, including the secondary somatosensory cortex, premotor, posterior parietal, superior temporal, and prefrontal cortices. The absence of a stimulation-related downstream activation beyond S1 as well as the functional disconnection of S1 from higher-order cortices were interpreted as signs of a limited capacity to process painful stimuli in patients with UWS/VS, showing a mere subcortical activation associated with a non-specific arousal response to acute pain [50]. A later study using a similar protocol found a pattern of residual cortical pain matrix involvement albeit without activation in the thalamus, prefrontal cortex and posterior parietal cortex, thus confirming the functional disconnection of single components within the cortical pain-related framework [51]. A further study investigated differences in brain activity in response to noxious stimuli among healthy controls and patients with UWS/VS or MCS [52]. Patients with UWS/VS showed activation in the thalamus and S1 but with greatly reduced functional connectivity as compared to healthy subjects. Striking neuroimaging differences were found between patients with UWS/VS and MCS although the stimulation did not elicit behavioural responses in either of the two patient groups. MCS patients showed activation across the pain matrix similar to that seen in healthy controls, although the pattern of activation was more lateralised and with less spatial extent [52]. Moreover, as compared to patients in UWS/VS, patients in MCS also showed a preserved functional connectivity between S1 and a wide network of associative areas, including the frontoparietal areas implicated in the conscious perception of external somatosensory stimuli [52]. These findings suggest that the perception of pain increases with the level of consciousness and that patients with MCS can experience pain to some extent even if, from a behavioural perspective, they cannot consistently or reliably communicate whether they are in pain [42,53].

A later case series focused on the potential prognostic value of somatosensory evoked potentials (SEPs) and functional magnetic resonance imaging (fMRI) during noxious stimulation [54]. The results confirmed the relationship between SEPs and fMRI findings and supported their proposed combined prognostic value as “a sort of neurophysiological Glasgow Coma Scale (GCS) score,” where surrogate measures of the residual integrity of the pain matrix are used as an indication of the potential for consciousness recovery [54]. In this respect, the most useful findings to disentangle the mechanisms of pain perception in patients with UWS/VS have come from a large sample fMRI study involving 30 patients with a diagnosis of non-traumatic UWS/VS and 15 healthy participants [55]. In this study, all of the participants were investigated through an alternating block design consisting of a standardised series of noxious electrical stimuli to the participants’ left index fingers alternated with a resting baseline condition. In the healthy group, the noxious stimulation significantly activated the S1 and S2, the ACC, the inferior frontal gyrus, the insula, the thalamus and the cerebellum. The activation pattern was less homogenous in the UWS/VS group, where 50% of the patients showed a significant activation in the sensory part of the pain matrix and/or the cerebellum, followed by a minority of patients showing a significant activation in the affective part of the pain matrix including the ACC and/or the anterior insula (30% of patients) or in both the sensory and the affective components (26.7% of patients) [55]. Activation in the lower order structures was found in half of the UWS patients while higher order structures were activated in four patients only. The results of this study are particularly relevant as they further highlight the possibility that pain perception may be underestimated through clinical assessment alone. Moreover, these findings focused the attention on the possible role of the cerebellum, traditionally not considered part of the pain matrix, in the complex process of pain perception both in healthy subjects and in patients with DOCs. However, a better understanding of the mechanisms underlying the conscious perception of pain would benefit from additional connectivity analyses among the involved areas. Future studies should take into account this issue in order to better disentangle the neural correlates of pain perception and to help avoiding misdiagnoses across different groups of patients.

Further studies have investigated the neural correlates of empathic responses in patients with DOCs. In healthy individuals, listening to pain cries has been reported to elicit functional activation within the pain matrix of the brain, so several studies have used this task to assess affective consciousness in behaviourally unresponsive patients. In a recent study, brain haemodynamic responses to pain cries, compared with neutral human vocalisations, were investigated through fMRI in a large sample of patients with UWS/VS [56]. The final findings showed that the pain matrix was activated by pain cries in more than half of the investigated patients. As the authors pointed out, although this does not prove subjective experience of emotion in relation to the stimulus, far less cognitive empathy, it does support the idea of an affective consciousness which is more fundamental than cognitive consciousness and for which the neural prerequisites may be more enduring in the face of severe cerebral damage [56].

A similar experimental paradigm was used by a further study to improve accuracy in differentiating patients in UWS/VS and MCS [57]. On exposure to sounds of human pain and suffering, no significant differences were shown between UWS/VS and MCS groups through task-related fMRI. However, in contrast with this finding, the weighted global connectivity was significantly greater in the MCS group compared to the UWS/VS group. Specifically, extensive functionally connected networks were seen in MCS patients, somewhat similar to those in healthy controls, in contrast with very limited and localised connectivity in the UWS/VS group. Thus, the authors proposed the analysis of the brain’s global functional connectivity as a potentially useful tool for reliably differentiating between UWS/VS and MCS [57]. Finally, other studies investigated pain perception in patients with DOCs by implementing specific neurophysiological approaches mainly based on the use of laser-evoked potentials [58,59,60,61,62,63,64,65]. The most comprehensive results came from a study investigating the presence of both the Aδ-fiber and the C-fiber laser evoked potentials as a marker of a residual cortical pain processing [62]. This study confirmed the presence of the previously mentioned difference between patients in UWS/VS and MCS, as the former showed increased latencies and reduced amplitudes of both the Aδ-LEP and C-laser evoked potential components as compared to the latter. Moreover, some patients in UWS/VS were reported to have only the C-laser evoked potential components, thus suggesting that the cortical generators of these components are more likely to survive a severe brain injury and may represent a useful tool for instrumental pain assessment in the most damaged patients [62]. All together, these findings pave the way for the identification of potential neurophysiological markers of conscious pain perception in behaviourally unresponsive patients and contribute to a better differential diagnosis between UWS/VS and MCS. It follows that a combination of clinical, neuroimaging and neurophysiological markers may represent the best way to disentangle the issue of pain perception and processing in patients with DOCs.

### 2.1. Neuroimaging Evidence to Optimise Treatments and Rehabilitation

Ultimately, a more comprehensive knowledge about brain functioning can help to personalise rehabilitation by recognising the patient’s strengths and limitations. This may include identifying possibilities for therapies using neuromodulation, with neuroimaging being used also in the monitoring of changes in time within the same patient and evaluating the effectiveness of treatment strategies [25,66,67]. Some therapeutic approaches have in fact been reported to speed the recovery of consciousness in subgroups of patients in UWS/VS or MCS [68,69,70,71,72,73,74,75,76]. These approaches include the use of pharmacological agents, such as zolpidem, levodopa, dopamine agonists and intrathecal baclofen as well as non-pharmacological therapies including deep brain stimulation, transcranial magnetic stimulation, transcranial direct current stimulation and spinal cord stimulation [68,69,70,71,72,73,74,75,76]. Within this framework, neuroimaging techniques could help to better identify which brain pathways are viable and non-viable in patients who are behaviourally unresponsive, enabling the planning of tailored therapeutic approaches to increase the chances of functional recovery. In reality, neuroimaging cannot yet be routinely used in such a way in clinical practice due to cost, availability, procedural complexity and the need to further optimise and validate these techniques as an evidence-based tool. In time, however, it may play an important role in profiling behaviourally unresponsive patients on the basis of their concomitant neurological impairments and comorbidities [33]. If targeted interventions for patients can encourage the recovery of even minimal purposeful behaviours, these patients may be enabled to communicate their needs and any sources of discomfort or pain.

### 2.2. Interpreting the Findings and Looking to the Future

A degree of caution is, of course, required in the interpretation of neuroimaging findings [77]. Both pain and consciousness are internal, subjective phenomena and neuroimaging, however sophisticated, cannot reveal the qualitative nature of a first-person experience. It can only provide us with quantitative surrogate measures. There is also an inherent imbalance in the conclusions that can be drawn from such research, since consciousness can be satisfactorily demonstrated empirically while its absence cannot be irrefutably proven and the same can be said about pain [78]. Moreover, there may be a difference between what occurs in the experimental setting and the physiological or pathological features of a real pain experience. In fact, experiments using nociceptive stimuli in patients with DOCs have to satisfy specific ethical principles that prevent patients being exposed to sources of significant discomfort. This introduces a potential bias in this field of research, as the intensity of the experimental stimuli is usually not comparable to that of real pain experienced by patients in pathological conditions (for instance when an acute pulpitis or a renal colic occurs). This fact is a serious limitation for the interpretation of all negative findings. Moreover, the processing of a time-limited and specific experimental stimulus, such as an isolated electrical stimulation, only partly resembles the processing of naturally occurring internal sources of pain, which could include mechanisms of sensitisation leading to the development of chronic pain [42]. In this respect, there is the possibility that, in patients with DOCs, pain can become a disorder in itself, persisting even when the original triggering pathology has been eradicated [39]. This could result through the development of chronic and centralised pain, favoured by maladaptive neural processes during the long clinical course of the disease [39].

When interpreting findings about the degree of both consciousness and pain perception in behaviourally unresponsive patients, it should be taken into account that diagnostic processes may vary between centres, and it is important that clinical descriptions and diagnostic criteria used are reported [79]. Researchers can select patients from the extreme ends of scores on responsiveness, highlighting clear group differences between UWS/VS and MCS. Those patients around the borderline between the two, or in an incremental transition, may be under-represented. This may contribute to somewhat invalidate the conclusions of the studies or to limit the results to confined subgroups of patients as a result of their wide heterogeneity and of the within-subject variability across time. To avoid misinterpretations and misdiagnoses, a combination of behavioural, neuroimaging and neurophysiological assessment is strongly encouraged. Moreover, behavioural observation has the advantage that the relatives of the patient can participate in an ongoing way, alerting clinicians to perceived behavioural changes in the patient, which may not have been present at the moment of formal clinical assessment. This applies to pain behaviours but also to responsiveness in general. However, if the ultimate aim is for neuroimaging to be incorporated widely as a tool for optimising individual care plans, more focus is required on single-subject studies, including longitudinal studies to map functioning and track changes in individual brains over time [25,57]. In fact, it has been highlighted that a severely damaged brain will not necessarily be comparable to a healthy brain [25] or to other injured brains in terms of structural functioning, which will vary depending on the type and location of injury sustained [80]. The development of techniques appropriate to patients with DOCs and single-subject studies is a challenge being addressed through both scanning protocols and methods of analysis [25,52,57]. On the other hand, large sample studies are also necessary to allow comparisons among different groups of patients. In fact, the findings of group analyses in the mentioned studies confirm the wide heterogeneity of patients with respect to the pathophysiology, extent and localisation of the underlying brain damage and the time elapsed from the primary injury. For instance, a trend to more frequent activations of the pain matrix in (sub)acute UWS/VS patients as compared with chronic UWS/VS patients has been observed [55]. The activation of isolated islands of the pain matrix in chronic patients is associated with signs of general cortical atrophy of various degrees with widening of the inner and outer cerebrospinal fluid spaces [51]. Opposite findings have been reported in studies investigating residual affective consciousness and showing equally distributed brain activation patterns among patients with a different disease duration [56]. Moreover, given the high number of comorbidities previously described in these patients, it would be useful to correlate pain-induced activation patterns with the comorbidity profile of individuals patients, in order to better clarify how the number and the severity of long standing comorbidities may influence the development of central sensitisation mechanisms leading to pain hypersensitivity. This would help to determine whether normal inputs, including those that usually evoke innocuous sensations, may represent sources of discomfort in patients with DOCs [81]. If the presence of sensitisation phenomena were confirmed, it should be expected that some patients with DOCs could experience a condition of continuous pain. This would invalidate the findings of those studies using a research protocol based on the alternation between pain stimuli and rest conditions. 

## 3. Conclusions

Neuroimaging is contributing to our understanding of the substrates of consciousness and pain perception in patients with DOCs. Findings suggesting partial brain activation within the pain matrix even in patients with persistent UWS/VS caution against facile assumptions about the nonsentient state of behaviourally unresponsive patients. On the other hand, such evidence does not allow us to conclude that the perception of pain in these patients resembles the constellation of pain experiences of healthy subjects. Moreover, it cannot be excluded that pathological and chronic pain mechanisms may develop along the prolonged clinical course of the disease due to the engagement of pathological neuroimmune pathways [82]. In MCS patients, the combination of significant levels of localised brain activation and a pattern of functional connectivity similar to healthy controls, albeit less extensive, point to the likelihood of a more functional pain perception. The activation of the anterior cingulate cortex in some patients implies that this might include an affective component.

What cannot be concluded from the evidence is that any individual patient with a DOC does not have any sentient experience of pain. Given the remaining uncertainty about this issue, a “just in case” approach to analgesics is advocated on ethical grounds [80], with medication provided in line with the nature of injuries, the patient’s pain history and the medical interventions necessary. It is pointed out that cautionary analgesia according to the patient’s physical condition may also protect against harm from the chronic effects of hormonal and immune reactions to nociception with or without perceived pain. Future clinical and neuroimaging studies are necessary to improve our knowledge about the intriguing relationship between consciousness and pain perception both in healthy subjects and in severely brain-injured patients. Given the practical and emotional importance of this issue to those involved, the scientific community has an obligation to develop the best possible means of assessing pain perception and to periodically review the evidence.

## Figures and Tables

**Table 1:** Main neuroimaging studies investigating pain perception in patients with disorders of consciousness.

Authors, Year	Number of Subjects	Clinical Diagnosis	Instrumental Assessment	Nociceptive Stimulus	Main Findings
Laureys et al., 2002 [50]	15 patients	UWS/VS	PET scanning	High-intensity electrical stimulation of the median nerve	Overall cerebral metabolism was 40% of normal values.
					Pain-induced activation of midbrain, contralateral thalamus, and contralateral primary somatosensory cortex (S1).
					Functional dissociation of S1 from higher-order associative cortices.
Kassubek et al., 2003 [51]	7 patients	UWS/VS	PET scanning	High-intensity electrical stimulation of the median nerve	Widespread marked overall hypometabolism.
					Pain-induced activation of contralateral primary somatosensory cortex (S1), secondary somatosensory cortex (S2), cingulate cortex and ipsilateral posterior insula.
Boly et al., 2008 [52]	20 patients compared to 15 healthy subjects	UWS/VS (15) MCS (5)	PET scanning	High-intensity electrical stimulation of the median nerve	VS patients: pain-induced activation of midbrain, contralateral thalamus, and contralateral primary somatosensory cortex (S1).
					MCS patients: pain-induced activation of contralateral thalamus, primary somatosensory cortex (S1), secondary somatosensory cortex (S2), inferior parietal lobule, superior temporal gyrus, dorsolateral prefrontal cortex and medial anterior cingulate cortex.
					Healthy subjects: pain-induced activation of ipsilateral and contralateral thalamus, contralateral primary somatosensory cortex (S1) and secondary somatosensory cortex (S2), ipsilateral and contralateral inferior parietal lobule and superior temporal gyrus, ipsilateral and contralateral striatum and dorsolateral prefrontal cortex and medial anterior and posterior cingulate cortex.
Zanatta et al., 2012 [54]	3 patients	Coma	Combination of fMRI and somatosensory-evoked potentials	Bilateral electrical stimulation of the median nerve	Presence of preserved middle latency evoked potentials combined with evidence of cortical activation at fMRI are good predictors of consciousness recovery.
Markl et al., 2013 [55]	30 patients compared to 15 healthy subjects	UWS/VS	fMRI	Electrical stimulation of the left index finger	Healthy subjects: pain-induced activation of the primary somatosensory cortex (S1) and secondary somatosensory cortex (S2), the anterior cingulate cortex (ACC), the inferior frontal gyrus, the insula, the thalamus, and the cerebellum.
					UWS/VS patients: pain-induced activation of the sensory-discriminative pain network in 50% of patients and of the affective pain network in 30% of patients.
Yu et al., 2013 [56]	44 patients	UWS/VS	fMRI	Pain cries from other people	Activation of the pain matrix areas in 24 patients (activation of the sensory subsystem in 34% of patients and of the affective subsystem in 30% of patients).
Kotchoubey et al., 2013 [57]	12 patients compared to 17 healthy subjects	UWS/VS (6) MCS (6)	fMRI and analysis of weighted global connectivity	Pain cries from other people	No significant differences in functional activation between the UWS/VS and MCS groups in task-related fMRI; greater weighted global connectivity in the MCS group compared to the UWS/VS group.
De Tommaso, 2013 [58]	7 patients compared to 11 healthy subjects	UWS/VS (3) MCS (4)	Laser and somatosensory evoked potentials recording; auditory mismatch negativity	Laser and electrical stimulation; auditory paradigm	Presence of laser evoked potentials in all the patients with a significant N2 and P2 latency increase.
					Absence of late somatosensory potentials in all the patients with the exception of one MCS patient, showing a significant N2 and P2 latency increase.
					Presence of auditory mismatch negativity in all the patients.
Naro, 2015 [59]	10 patients compared to 10 healthy subjects	UWS/VS	Combination of motor evoked potentials and laser evoked potential to investigate pain-motor integration	Laser stimulation	No significant differences in the resting motor threshold between UWS/VS patients and healthy subjects; significantly compromised pain-motor integration in UWS/VS patients as compared to healthy subjects with some patients showing signs of partially restored pain-motor integration.
De Salvo, 2015 [60]	23 patients	UWS/VS (13) MCS (10)	Laser evoked potentials (LEP) recording	Laser stimulation	Lower LEP amplitudes and more delayed LEP latencies in patients in UWS/VS as compared to patients in MCS.
De Tommaso, 2015 [61]	9 patients compared to 11 healthy subjects	UWS/VS (5) MCS (4)	Laser, somatosensory, auditory and visual evoked potentials recording	Laser stimulation	Variable degree of preservation of evoked responses in UWS/VS patients as compared to healthy subjects, with the exception of laser evoked potentials that were recognized in all the patients.
Naro, 2015 [62]	38 patients compared to 15 healthy subjects	UWS/VS (23) MCS (15)	Aδ-fiber laser evoked potentials (Aδ-LEP) and C-fiber laser evoked potentials (C-LEP) recording	Laser stimulation	Higher LEP amplitudes and less delayed LEP latencies in healthy subjects as compared to DOC patients.
					Higher LEP latencies in patients with UWS/VS as compared to patients with MCS, no significant differences in LEP amplitude.
Aricò, 2016 [63]	14 patients	UWS/VS (8) MCS (6)	LEP recording and 24 h-polysomnography	Laser stimulation	Higher LEP latencies and lower LEP amplitudes in patients with UWS/VS as compared to patients with MCS.
					More preserved sleep-wake cycles and a more structured sleep in patients with MCS as compared to patients with UWS/VS.
Naro, 2016 [64]	33 patients	UWS/VS (18) MCS (15)	Evaluation of Repetitive Laser Stimulation-induced gamma-band oscillation (GBO) power and clinical assessment through the NCS-R	Repetitive Laser stimulation	Increase in GBO power and NCS-R score in all the MCS patients.
					No significant increase in GBO power and NCS-R score in the UWS/VS group with the exception of five patients.

UWS: unresponsive wakefulness syndrome; VS: vegetative state; MCS: minimally conscious state; PET: Positron Emission Tomography; fMRI: functional Magnetic Resonance Imaging; LEP: Laser Evoked Potentials.
